# Muscle protein breakdown is impaired during immobilization compared to during a subsequent retraining period in older men: no effect of anti-inflammatory medication

**DOI:** 10.1007/s00424-020-02353-w

**Published:** 2020-02-05

**Authors:** K. Dideriksen, S. Reitelseder, J. Agergaard, A. P. Boesen, S. N. Aas, T. Raastad, Lars Holm

**Affiliations:** 1 Institute of Sports Medicine Copenhagen, Department of Orthopedic Surgery M, Bispebjerg Hospital and Center for Healthy Aging, Faculty of Health and Medical Sciences, University of Copenhagen, Copenhagen, Denmark; 2 Department of Biomedical Sciences, Faculty of Health and Medical Sciences, University of Copenhagen, Copenhagen, Denmark; 3 Department of Physical Performance, Norwegian School of Sport Sciences, Oslo, Norway; 4 School of Sport, Exercise and Rehabilitation Sciences, University of Birmingham, Edgbaston, Birmingham, B15 2TT UK

**Keywords:** Deuterated water, Deuterated alanine, Muscle degradation, Muscle disuse, Muscle recovery, NSAID

## Abstract

Muscle inactivity reduces muscle protein synthesis (MPS), whereas a subsequent period of rehabilitation resistance training (retraining) increases MPS. However, less is known regarding muscle protein breakdown (MPB) during such conditions. Furthermore, nonsteroidal anti-inflammatory drugs (NSAIDs) may have a dampening effect on MPB during periods of inactivity in older individuals. Thus, we measured the average MPB, by use of the deuterated water methodology, during an immobilization period and a subsequent retraining period in older individuals with and without NSAID treatment. Eighteen men (60–80 years: range) were randomly assigned to ibuprofen (1200 mg/d, Ibu) or placebo (Plc). One lower limb was immobilized in a cast for 2 weeks and retrained for 2 weeks, and 2 × 20 g of whey protein was ingested daily during both periods. Besides MPB, the protein expression of different muscle degradation signaling molecules was investigated. MPB was lower during immobilization compared to retraining (*p* < 0.01). NSAID treatment did not affect the MPB rate during immobilization or retraining (*p* > 0.05). The protein expression of muscle degradation signaling molecules changed during the study intervention but were unaffected by NSAID treatment. The finding that MPB was lower during immobilization than during retraining indicates that an increased MPB may play an important role in the muscle protein remodeling processes taking place within the initial retraining period. Moreover, NSAID treatment did not significantly influence the MPB rate during 2 weeks of lower limb immobilization or during 2 weeks of subsequent retraining in older individuals.

## Introduction

Disuse of skeletal muscle either in the form of reduced use [7], bed rest [13, 14], or immobilization [11, 41] causes atrophy. While it is well-established that the muscle protein synthesis rate declines during immobilization, the role of muscle protein breakdown (MPB) in relation to inactivity-induced muscle atrophy is less clear. So far, only few attempts have been made to measure MPB after periods of muscle inactivity. By use of the arteriovenous balance model in combination with stable isotope infusion, it has been demonstrated that the MPB is unchanged after 14 days of bed rest in young men [14]. Furthermore, through pulse isotope infusions, it has been shown that the muscle fractional breakdown rate (FBR) is unchanged after 21 days of bed rest in young men [42]. However, another study reported that interstitial 3-methylhistidine, a biomarker of myofibrillar protein breakdown, was increased with 3 days of immobilization in young men [44]. Taken together, these findings indicate that the MPB may variate throughout the period of muscle inactivity with a transient elevation during the early inactivity period followed by a return to baseline levels during sustained periods of inactivity. However, the findings obtained with the tracer dilution methods provide a nonprotein-specific MPB measurement, and moreover, all reported values represent acute “snap shots” of the MPB rate. Therefore, these findings may not be fully representative of the MPB during daily life periods of immobilization. Although the tracer dilution methods can be advantageous in relation to measurement of net tissue balance with a high time resolution, the deuterated water methodology provides a protein specific and direct measurement of the average MPB over a period of daily living (days) [18].

Conduction of muscular contractions, e.g., resistance exercises, stimulates muscle protein turnover rates in the following hours/days of recovery [3, 30]. Moreover, muscle protein synthesis has been shown to be elevated during the first 8 days of resistance exercise training [50]. However, less is known regarding the specific fluctuations in MPB during prolonged periods with repeated resistance exercise sessions, although it seems possible that the MPB would increase due to the processes of skeletal muscle remodeling that occurs during the early period of unaccustomed resistance exercise [9]. Especially, the early period of rehabilitation resistance training, after a period of muscle inactivity, could be expected to have a significant impact on the overall muscle protein turnover and hence also muscle MPB. Nevertheless, as compared to the state of inactivity, early rehabilitation resistance training represents the complete opposite state, and hence, the two conditions make up two “extremes” within the normal life span of most people.

It has been demonstrated that NSAIDs may influence the muscle mass adaptation to periods of resistance training in healthy, older humans [45], as a consequence of alterations in muscle protein turnover kinetics [46]. Especially, the observation that NSAIDs inhibit the training induced increase in muscle gene expression of interleukin-6 (IL-6) and muscle RING finger protein 1 (MuRF-1) indicates that MPB may be affected by NSAID intake [46]. Moreover, supplementation with dietary omega-3 fatty acids (which can have anti-inflammatory effects [2, 16]) prior to and during 10 days of immobilization has been shown to alleviate muscle catabolism in healthy, adult rats [51]. This muscle-preserving effect was partly achieved by eliminating the increases in the muscle expression of the ubiquitin ligases, MuRF1 and Atrophy gene-1 (Atrogin-1) [51], which regulate muscle degradation via the ubiquitin-proteasome system [27, 35]. Somehow in line with that, it has been demonstrated that protein feeding induces an increase in Forkhead box O (FoxO)-3a phosphorylation, alongside a decrease in muscle degradation, in elderly inflamed rats treated with NSAID, but not in placebo-treated rats [33]. Taken together, these findings could indicate that anti-inflammatory treatments may have a diminishing effect on MPB in both catabolic and anabolic situations such as muscle inactivity, protein ingestion, and resistance training.

Even though the effect of NSAID on both muscle gene and protein signaling response to resistance exercise has been investigated [25, 46, 48], the information is still sparse. The nuclear factor-kappa B (NF-κB) transcriptional activator is regarded as a central regulator of muscle inflammatory signaling pathways that can induce transcription of the atrogens, which may ultimately lead to increased muscle degradation [20, 21, 36, 48]. Upon activation of the NF-κB complex, regulatory molecules, which keep the NF-κB transcription factors sequestered in the cytosol, are phosphorylated and removed, which leads to the transport of NF-κB transcription factors into the nucleus [20, 21, 36, 48]. Therefore, the relationship between the concentrations of NF-κB transcription factors, measured in the cytosol and the nucleus subcellular fractions, respectively, could give an indication of the NF-κB activation. Interestingly, the observation that NSAIDs may inhibit the training induced increase in MuRF-1 gene expression [46] has led to speculations of that this may occur through a pathway involving both prostaglandin (PG)E2 and NF-κB [38, 46]. However, other work could not clearly demonstrate that NSAID affected the activation NF-κB [48], and more work is needed to further clarify this.

The present study aimed to investigate the rate and intramyocellular regulation (protein signaling) of the MPB process during 2 weeks of lower limb immobilization and 2 weeks of subsequent retraining. Since immobilization may induce some degree of local muscle inflammation [40], NSAID treatment was applied to dampen local muscle inflammation during a period of immobilization and retraining. The deuterated (^2^H_2_O) water–based method was used to assess the gross average myofibrillar fractional breakdown rate, as previously described [18]. Furthermore, protein concentrations of signaling molecules related to MPB were measured both in the cytosolic and nuclear subcellular fractions at baseline, immediately after immobilization, 20 h after the first retraining session and after 2 weeks of retraining. We assumed that prolonged inactivity obtained by immobilization would decrease muscle protein turnover rates (also MPB), and therefore, it was hypothesized that MPB would be lower compared to during the subsequent retraining period. Furthermore, it was hypothesized that treatment with NSAID would have a dampening effect on MPB during immobilization but that it would not influence the retraining conditions.

## Methods

### Study design

#### Experimental protocol

Eighteen healthy, older males (age, 60–80 years; BMI, 20–30 kg/m^2^) were recruited through newspaper advertisements. The included subjects had no cancer and metabolic, cardiac, or neurological diseases; were nonsmokers and recreationally physically active; and had not taken part in any form of strenuous endurance or resistance training before trial participation. Furthermore, subjects gave their written informed consent before being enrolled in the experiment that were approved by the Copenhagen Ethics Committee (H-1-2010-007) and conformed to the Helsinki Declaration. Finally, subjects were instructed not to take any kind of analgesic medication at least 2 weeks before the beginning of the study.

**Fig. 1:**
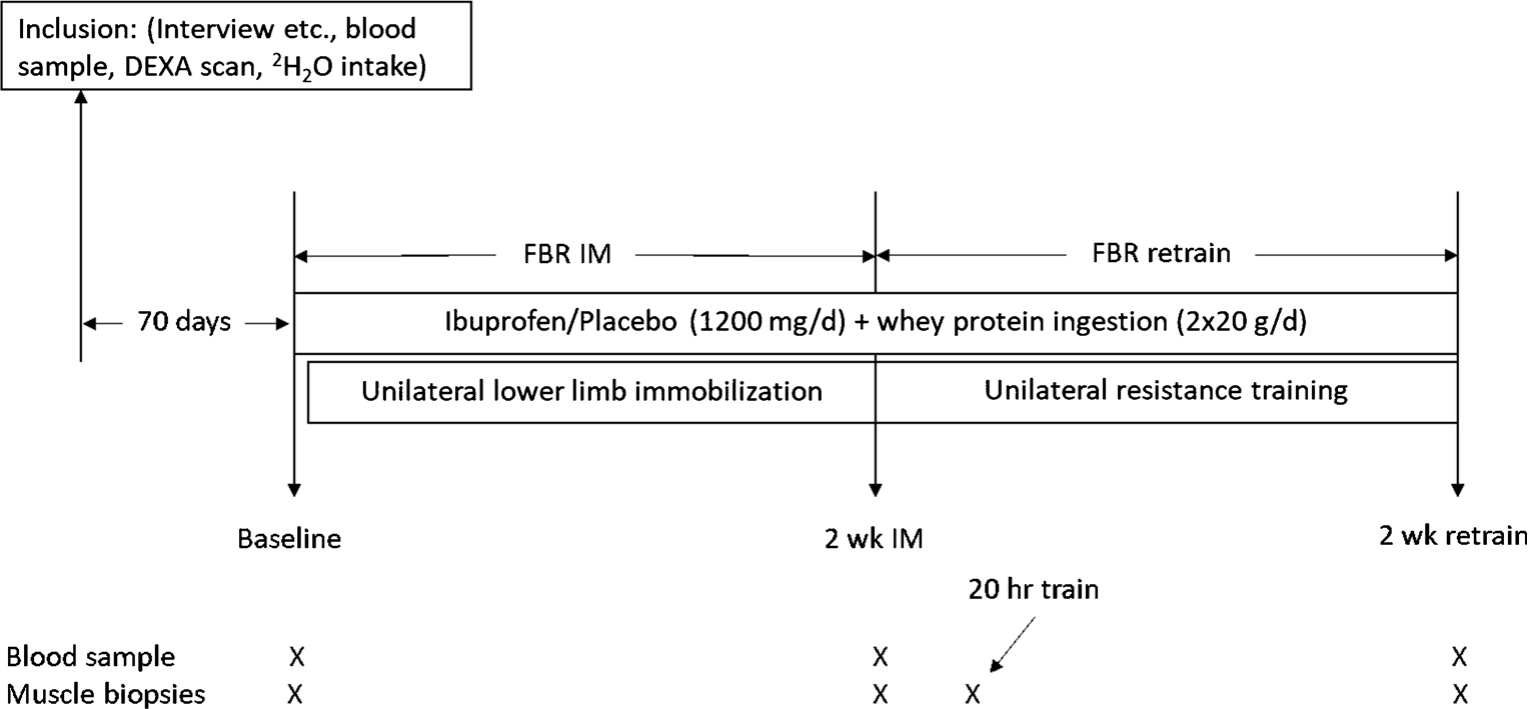
Study intervention protocol. 18 older men completed 2 weeks of immobilization (2-week IM) and 2 weeks of retraining (2-week retrain) during which period they all ingested 2 × 20 g of whey protein per day. The participants were randomized into groups double blindly receiving either ibuprofen (*n* = 8) or placebo (*n* = 10) administration (2 × 600 mg/day). Measurements were performed at baseline, after 2-week IM, after the first retraining session (20-h retrain), and after 2 weeks of retraining (2-week retrain)

The experimental design is shown in Fig. 1. The present investigation was a part of another experiment that investigated the effect of ibuprofen treatment on muscle mass and strength adaptation during 2 weeks of lower limb immobilization and 6 weeks of subsequent retraining [11]. Moreover, the subject characteristic data of the included individuals have been used in previous studies [4, 11, 12], whereas all other presented data have not been reported previously.

#### Ibuprofen treatment and protein supplementation

The included individuals were divided into 2 groups receiving either placebo (Plc, *n* = 10) or ibuprofen (Ibu, *n* = 8) in a randomized and double-blinded fashion. As previously described [11], the placebo group consists of 2 groups. The first group of 4 subjects has been included in a previous study [4] and received double-blinded placebo injections (placebo or growth hormone), whereas the remaining 6 subjects in the placebo group received placebo tablets in a double-blinded fashion (placebo or ibuprofen). All subjects completed the study intervention during the same time period.

The ibuprofen treatment started 2 days before the immobilization period to ensure that all subjects were fully treated from the onset of immobilization. The placebo tablets contained mainly potato starch and lactose monohydrate and were visually identical to the ibuprofen tablets. Moreover, all individuals were instructed to take their daily tablets (2 × 600 mg/day) at the same time every morning and evening together with a meal and to return the empty packages. Furthermore, subjects were not allowed to consume any cyclooxygenase-inhibiting drugs besides the tables provided during the study period.

The present study was carried out under free-living conditions, and all subjects were instructed to maintain their normal diets and to consume 2 × 20 g of whey protein (Lacprodan, Arla Foods Ingredients P/S, Viby J, Denmark) each day throughout the study period, as previously described [11]. This was done to ensure that eventual differences in myofibrillar FBR between groups would not be due to insufficient intake of protein and essential amino acids in one of the groups. Finally, both the ibuprofen treatment and the protein supplementation were initiated at baseline (day 70) and maintained for the rest of the study period.

#### Unilateral limb immobilization and retraining

The immobilization procedure has previously been described in detail and shown to induce substantial quadriceps muscle atrophy in both young and older individuals [4, 5]. Briefly, immobilization was accomplished by a lightweight fiber cast applied from the groin to just proximal to the malleoli (randomly selected limb). The cast was positioned at 50° of knee joint flexion to impede walking ability (0° corresponding to full extension). Furthermore, the subjects were carefully instructed to avoid any kind of quadriceps muscle contractions and to use crutches for locomotion. However, subjects were encouraged to remain physically active during the unilateral immobilization period, although it may cause them some difficulties. Moreover, all subjects were treated with acetylsalicylic acid (75 mg/day) during the 2-week immobilization period to reduce the potential risk of deep venous thrombosis. Even though acetylsalicylic acid is an NSAID, the anti-inflammatory effect of such a low dose was most likely negligible.

After removal of the cast, the subjects received supervised unilateral resistance training 3 times per week for 2 weeks (all individuals completed a total of 6 weeks of retraining, but only the 2 first weeks are included in the present investigation). After 5 min of warm-up on a stationary bike, the subjects performed knee extension and leg press in randomized order in training machines (Technogym, Gambettola, Italy). The training intensity and volume were 3–4 sets × 12 repetitions (15 repetitions maximum (RM)) in week 1 and 4 sets × 10 repetitions (12 RM) in week 2. The training load was adjusted each week by the use of 5 RM tests.

### Measurements

At inclusion, the whole-body lean body mass (LBM) was determined by dual-energy X-ray absorptiometry scanning (Lunar DPX-IQ, GE Healthcare, Chalfont St. Giles, UK). During the scan, the individuals were wearing light clothing, no removable metal objects, and placed in a supine position. Thereafter (at day 0), subjects drank a total volume of 5.25-mL/kg LBM^−1^ of 99% ^2^H_2_O, which was diluted 1:1 with tap water and provided in 2–3 boluses over a 1-h period. Venous blood samples were taken right before and 2 h after the deuterated water bolus was consumed, and again at baseline (day 70), after immobilization (day 86), and after 2 weeks of retraining (day 100) in order to determine the deuterium enrichment of serum-free alanine. Blood samples were drawn from the antecubital vein into clot activator tubes, which were allowed to clot at room temperature for 30 min and followed by centrifugation (10 min at 3970 g at 4 °C). Serum was stored (− 80 °C) for analyses of ^2^H-alanine enrichment.

At baseline, after immobilization, and after 2 weeks of retraining, the subjects met at the lab, were placed in a supine position, and had a muscle biopsy taken from the vastus lateralis muscle using local anesthesia (lidocaine, 1%) and a 5-mm Bergström needle with suction. The muscle biopsies were cleared of external adipose tissue, connective tissue, and blood; frozen in liquid nitrogen; and stored at − 80 °C for subsequent analysis. The muscle biopsies were taken from the non-immobilized limb at baseline and from the immobilized limb after immobilization and 2 weeks of retraining. The biopsies from the immobilized limb were placed at least 3 cm apart to obtain samples unaffected by previous biopsies, and the order of location was randomized. Furthermore, these three biopsies were used for measurement of both muscle protein-bound abundances of ^2^H-alanine and muscle degradation signaling. The individuals refrained from strenuous physical activity 72 h prior to biopsy sampling at baseline (day 70), whereas prior retraining sessions were performed approximately 48 h before the biopsy obtained after 2 weeks of retraining (day 100). Additionally, a muscle biopsy was taken from the immobilized limb 20 h after the first retraining session (at day 87), with the purpose to measure the muscle degradation signaling response to acute retraining. At baseline and after immobilization, the biopsies were taken in the fasted state, whereas a light meal was ingested in the morning of the day of biopsy sampling after the first retraining session and after 2 weeks of retraining (ingested 2–3 h before the biopsy sampling). Finally, all muscle biopsies were collected at the same time of the day (± 1 h) to avoid effects of circadian variations on muscle degradation signaling.

### Analyses

#### Serum-free alanine preparation

Serum ^2^H-alanine enrichment was measured in 200-μL serum. The samples were acidified with 1-mL 50% acidic acid and poured over resin columns (AG 50 W- × 8 resin; Bio-Rad Laboratories, Hercules, CA) preconditioned with 1-mL 50% acidic acid. After 5 washes with Millipore water, the purified amino acids were eluted with 2 × 1 mL of 2-M NH_4_OH. After being dried down under a stream of nitrogen, the purified amino acids were derivatized using MtBSTFA + 1% tBDMCS (Regis Technologies, Inc., Morton Grove, IL, USA) and acetonitrile with a volume relation of 1:1. The tBDMS derivatives of the amino acids were separated by gas chromatography (GC) on a 30-m CP-Sil 8 CB capillary column (ChromPack, Varian, Palo Alto, CA) using programmed temperature vaporization (PTV) mode and injecting approximately 1 μL. The alanine derivatives were analyzed by tandem mass spectrometry (MS) using a Thermo Scientific, TSQ Quantum GC-MS/MS (San Jose, CA, USA), operating in the electron ionization mode, as previously described [19].

#### Muscle myofibrillar protein-bound alanine preparation

Muscle specimens of 10–20-mg wet weight were homogenized (Fast-prep, 120A-230; Thermo Savant, Holbrook, NY, USA) for 2 × 45 s in 1.5 ml of ice cold Milli-Q saline water. After a spin (5 °C, 5.500 g, 10 min), the pellet was added 1 ml of homogenization buffer (0.02 M Tris, pH 7.4, 0.15 M NaCl, 2 mM EDTA, 0.5% Triton-X 100, and 0.25 M sucrose), homogenized for 2 × 45 s (Fast-prep), and left for 3 h at 5 °C. After a spin (5 °C, 800 g, 20 min), 1 ml of a homogenization buffer (0.02 M Tris, pH 7.4, 0.15-M NaCl, 2-mM EDTA, 0.5% Triton-X 100, and 0.25-M sucrose), homogenized for 45 s, and left for 30 min at 5 °C. After a spin (5 °C, 800 g, 20 min), the pellet was added 1.5 ml of high-salt solution (0.7-M KCl, 0.1-M pyrophosphate), homogenized (vortexed), and left overnight at 5 °C. After vortex and spinning (5 °C, 1600 g, 20 min), the supernatant (containing the myofibrillar fraction) was added 3.45-ml ethanol (99%), vortexed, and left for 2 h at 5 °C. After spinning (5 °C, 1600 g, 20 min), 1 ml of 70% ethanol was added to the pellet, which was vortexed and spun (5 °C, 1600 g, 20 min), where after the pellet was hydrolyzed overnight in 1 ml of 6 M HCl at 110 °C. The liberated amino acids were purified over resin columns (as described above for serum-free alanine) and N-acetyl-n-propyl (NAP)-derivatized. The ^2^H abundance in human myofibrillar proteins was analyzed on a gas chromatograph-pyrolysis-isotope ratio mass spectrometer (GC-P-IRMS) system (GC Combustion III, Delta Plus XL; Thermo Finnigan, Bremen, Germany). As previously described [18], the NAP-derivatized samples were injected in the PTV mode with the GC inlet initially set at 45 °C for 1 min, where after it was elevated to 280 °C with 20 °C/min. The GC column (30 m × 0.25-mm film thickness, 1 μm DB-1701; J&W Scientific) was ramped from 45 to 280 °C, and all GC column effluent passed into a high-temperature reduction reactor (1450 °C), where the organic compounds were reduced to hydrogen gas and carbon monoxide before entering the IRMS.

#### Calculation of muscle myofibrillar protein FBR

Paired samples were obtained, and we were able to determine the FBR of each individual’s muscle myofibrillar protein fraction. By log transformation of the measured enrichment values, the slope, k_B_, of the ln(E) versus time curve was determined as a gross mean between adjacent biopsy time points (16 days for immobilization and subsequently 14 days of retraining) [18].

#### Western blot analyses

Muscle samples of 10–25 mg were homogenized using a plastic pestle and fractionated into a cytosolic and nuclear fraction using a commercial fractionation kit (ProteoExtract Subcellular Proteome Extraction Kit, 539,790, Merck, Darmstadt, Germany) according to the manufacturer’s procedures. Subsequently, protein concentrations on the cytosolic and nuclear fractions were quantified using a commercial microplate kit (Bio-Rad DC protein microplate assay, 0113, 0114, 0115, Bio-Rad Laboratories, Hercules, CA, USA).

Equal amounts of protein were loaded per well (20–30 μg) and separated by 4–20% gradient Mini-PROTEAN TGX Stain-Free Precast protein gels (4,568,093, Bio-Rad Laboratories). Electrophoresis was performed under denaturized conditions for 40–45 min at 200 V in cold Tris/Glycine/SDS running buffer (161–0732, Bio-Rad Laboratories). After gel electrophoresis, proteins were transferred onto a PVDF-membrane at 100 V for 30 min (Criterion^TM^ Blotter; Tris/Glycine buffer 161–0734, Bio-Rad Laboratories). Membranes were blocked at room temperature for 2 h in a TBS solution with 5% fat-free skimmed milk and 0.1% Tween 20 (TBS, 170–6435, Bio-Rad Laboratories; Tween-20, 437082Q, VWR International, Radnor, PA, USA; Skim milk, 1.15363.0500, Merck). Blocked membranes were incubated overnight at 4 °C with a primary antibody against ubiquitin (3933, Cell Signaling Technology, Danvers, MA, USA) diluted 1:1000. After incubation, membranes were washed and incubated at room temperature for 1 h with a secondary antibody diluted 1:3000 (7074, Cell Signaling Technology). After detection, membranes were stripped of primary and secondary antibodies using Restore Western Blot Stripping Buffer (21,059, Thermo Fisher Scientific, Rockford, IL, USA), blocked for 2 h at room temperature, and incubated at 4 °C overnight with antibodies against FoxO3a (2497, Cell Signaling Technology), NF-κB p65 (ab7970, Abcam, Cambridge, UK), and IκBα (ab32518, Abcam), diluted 1:1000, 1:500, and 1:400, respectively. Subsequently, membranes were incubated in secondary antibody (7074, Cell Signaling Technology). All antibodies were diluted in a 1% fat-free skimmed milk TBS solution added 0.1% Tween-20. Between stages, membranes were washed in 0.1% TBS-T. All samples were analyzed in duplicates, and bands were visualized using a HRP-detection system (Super Signal West Dura Extended Duration Substrate, 34,076, Thermo Fisher Scientific). Chemiluminescence was measured using a ChemiDoc MP System (Bio-Rad Laboratories), and band intensities were calculated with Image Lab (Bio-Rad Laboratories). The use of stain-free technology allowed normalization to tryptophan content in the membranes after transfer, as these gels have been added a 58-Da Trihalo compound that covalently binds to tryptophan residues in proteins when expressed to ultraviolet (UV) light.

#### Statistics

The sample size was calculated based on the hypothesis on the fractional breakdown rates. We estimated the relative difference in FBR between the two conditions as the following. In the immobilized and retraining states, we expected a reduction and increase of ~20%, respectively, from normal living/activity conditions. We used a value for normal living/activity conditions of 1.73 ± 0.49%/d (mean ± SD) [18], and hence, we estimated the sample size based on a power of 0.80, a significance level of 0.05, and a group difference of 0.7 ± 0.4%/d to a total of seven research participants in two independent groups.

Before statistical analysis, all data set were tested for normality. Data were analyzed using a two-way ANOVA with repeated measures and Student–Newman–Keuls post hoc tests were performed when significant overall effects were observed. Muscle protein signaling data were log transformed before the statistical analysis and presented as geometric means ± back-transformed SE. Data are reported as means ± standard error of mean (unless otherwise stated). Differences were considered significant when *p* < 0.05. The statistical software SigmaPlot v. 12.3 (Systat Software Inc., San Jose, CA, USA) was used for all statistical tests.

## Results

### Subject characteristic

Age, height, weight, and body mass index did not differ between groups. Body weight (kg) was 78 ± 3, 79 ± 3, and 79 ± 3 in the Plc group and 84 ± 3, 84 ± 4, and 84 ± 4 in the Ibu group at baseline, after immobilization, and after 2 weeks of retraining, respectively. For body weight, an interaction effect (*p* < 0.05) was observed, and the post hoc test indicated that body weight was higher (*p* < 0.05) after immobilization and 2 weeks of retraining in the Plc group, whereas it did not change in the Ibu group.

The subjects completed the immobilization and retraining periods without reporting any clinical problems. Subjects reported full compliance regarding the ibuprofen administration and protein intake, which was supported by measurements of blood ibuprofen concentration indicating that all subjects in the Ibu group took their medication regularly, as described previously [11]. Furthermore, the total training load and training intensity did not differ between the Ibu and the Plc group, as described previously [11].

Due to insufficient availability of muscle biopsy material, the myofibrillar FBR was measured in both the immobilization and retraining periods in 7 (of 8) and 6 (of 10) individuals in the Ibu and Plc groups, respectively. Further, at the time point of 20 h after the first retraining session, muscle degradation signaling could only be obtained in 6 (of 10) subjects in the Plc group and in 7 (of 8) subjects in the Ibu group. At all other time points, the muscle MPB signaling was measured in all the included individuals.

### Serum ^2^H-alanine enrichment

An overall time effect (*p* < 0.001) was found for the serum-free ^2^H-alanine enrichment (Table 1). Two hours after ingestion of deuterated water (day 0), a marked ^2^H-alanine enrichment appeared in both groups (*p* < 0.001). At day 70 (start of immobilization period), 86 (end of immobilization), and 100 (end of retraining period), the ^2^H-alanine abundance had disappeared and was not different from zero (*p* > 0.05).

**Table 1:** Serum ^2^H-alanine enrichment measured 2 h after ingestion of deuterated water (day 0), as well as at day 70 (start of immobilization), day 86 (end of immobilization/start of retraining), and day 100 (end of retraining)

Serum ^2^H-alanine enrichment	Plc	Ibu
Day 0	0.542 ± 0.033*	0.500 ± 0.015*
Day 70	− 0.009 ± 0.007	− 0.008 ± 0.015
Day 86	− 0.026 ± 0.015	0.010 ± 0.013
Day 100	− 0.010 ± 0.005	0.030 ± 0.023

Data were analyzed with a two-way repeated measure ANOVA: A time effect (p < 0.001) was observed. *Significant different from all other values (*p* < 0.001). Data are means ± SE

### Muscle myofibrillar protein-bound ^2^H-alanine and fractional breakdown rate

**Fig. 2:**
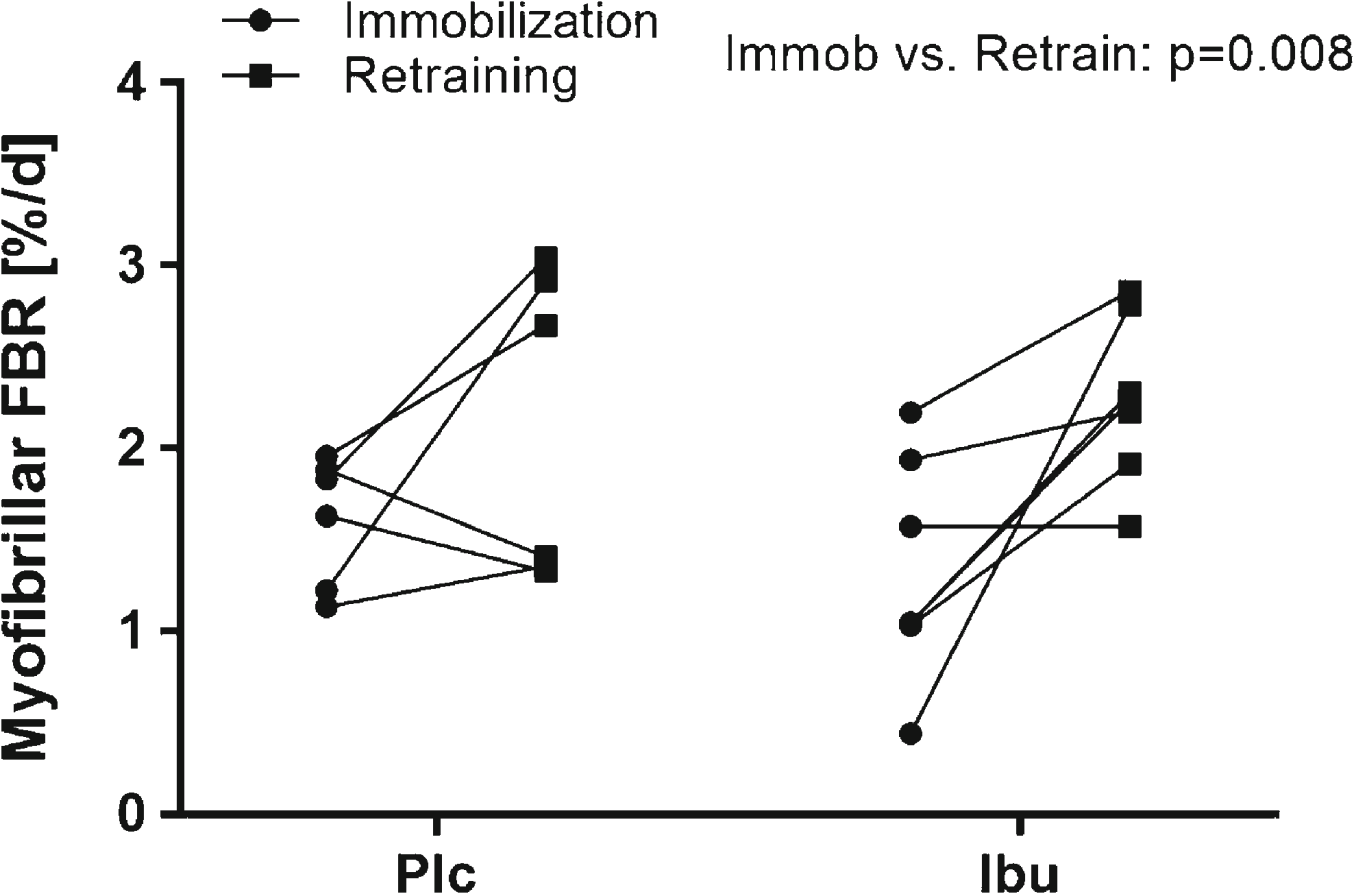
Muscle myofibrillar fractional breakdown rates (FBR) during 2 weeks of immobilization (• Immobilization) and 2 weeks of retraining (♦ Retraining). Data were analyzed with a two-way repeated measure ANOVA: A time effect (*p* < 0.01) was observed. Each dot represents the FBR of an individual, and connected dots are repeated measures during immobilization and retraining

An overall time effect (*p* < 0.01) was found for myofibrillar FBR (Fig. 2). The myofibrillar FBR was 1.61 ± 0.14%·d^−1^ during the immobilization period and 2.12 ± 0.34%·d^−1^ during the retraining period, in the Plc group. In the Ibu group, the myofibrillar protein FBR were 1.33 ± 0.23%·d^−1^ during the immobilization period and 2.27 ± 0.17%·d^−1^during the retraining period.

### Muscle protein breakdown signaling

**Fig. 3:**
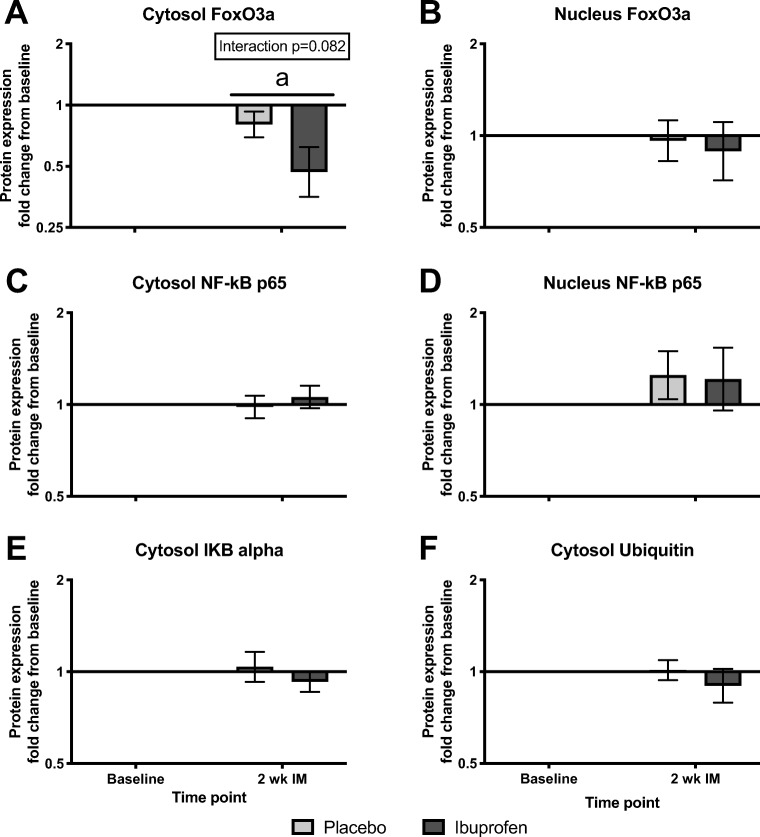
**a**, **b**, **c**, **d**, **e**, and **f**. Muscle proteolysis signaling at baseline and after 2 weeks of immobilization (2-week IM). Data are expressed relative to baseline levels and presented as geometric means ± back-transformed SE. Log-transformed data were analyzed with a two-way repeated measure ANOVA. No group or interaction effects were observed (*p* > 0.05), but time effects appeared (*p* < 0.05). **a** Denote significant different from baseline (*p* < 0.05). Tendency (0.05 < *p* < 0.10) from ANOVA testing is shown within figure in upper right panel

**Fig. 4:**
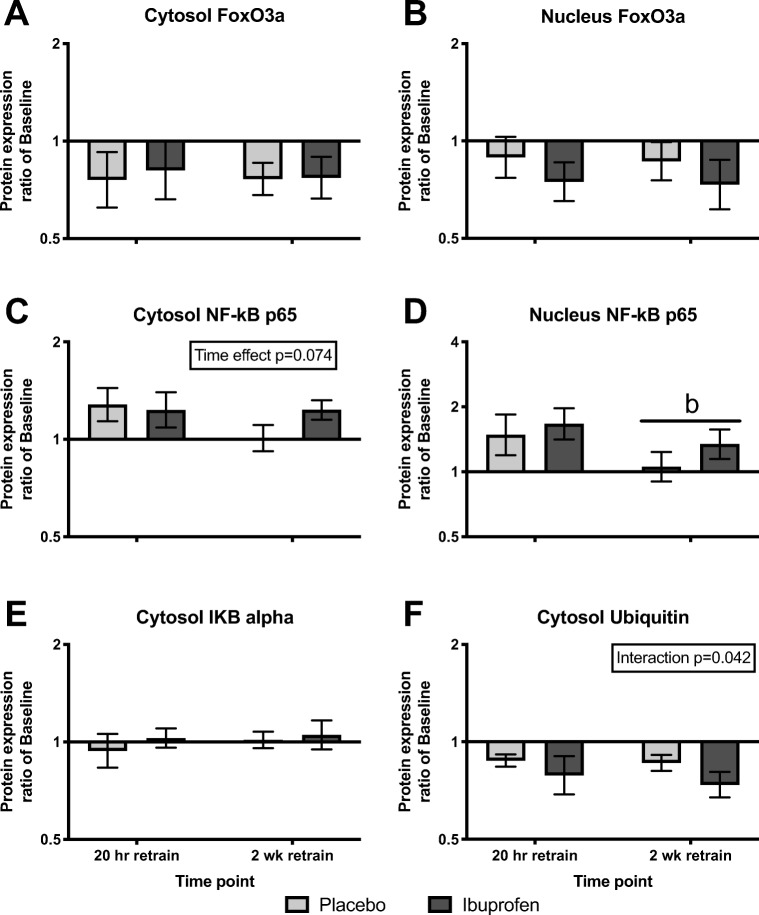
**a**, **b**, **c**, **d**, **e**, and **f**. Muscle proteolysis signaling after the first retraining session (20-h retrain) and after 2 weeks of retraining (2-week retrain). Data are expressed relative to baseline levels and presented as geometric means ± back-transformed SE. Log-transformed data were analyzed with a two-way repeated measure ANOVA. Interaction effect was observed in **f** (*p* < 0.05), but no significant differences from subsequent post hoc testing were seen. No group effects were observed (*p* > 0.05), but time effects appeared (*p* < 0.05). **b** Denote significant different from 20-h retrain (*p* < 0.05). Tendency (0.05 < *p* < 0.10) from ANOVA testing is shown within the figure in upper right panel

Muscle protein concentrations of signaling targets related to MPB are shown in Figs. 3 and 4. The biopsies pre- and post-immobilization (Fig. 3) were taken during overnight fasting conditions, whereas a standardized meal was ingested in the morning before muscle biopsy sampling during retraining (Fig. 4). Thus, the protein expressions related to immobilization and retraining, respectively were analyzed in separate statistical tests.

Among all the investigated protein targets, no group effects were found (*p* > 0.05). An effect of group x time (interaction) was found for cytosolic ubiquitin expression (*p* < 0.05), but subsequent post hoc tests did not reveal any significant differences (Fig. 4f). However, overall time effects were identified (*p* < 0.05), revealing a difference during immobilization and retraining, respectively. Results from the post hoc tests are described in the following sections.

#### FoxO3a concentration

The protein expression of FoxO3a in the cytosolic subcellular fraction (Fig. 3a) decreased after immobilization. During retrain, no differences were seen between the first retraining session and after 2 weeks of retraining (Fig. 4a). The nuclear FoxO3a protein expression was unchanged after 2 weeks of immobilization (Fig. 3b) and was not affected by retraining (Fig. 4b).

#### NF-κB p65 concentration

The cytosolic NF-κB p65 protein expression was unchanged after immobilization (Fig. 3c), and not significantly affected during retraining (Fig. 4c), although a tendency toward an effect of time was seen in the latter condition (*p* = 0.074). After immobilization, the nuclear NF-κB p65 expression was unchanged (Fig. 3d), whereas after the first retraining session, nuclear NF-κB p65 expression was greater than after 2 weeks of retraining (Fig. 4d).

#### Cytosolic IκBα and ubiquitinated protein concentrations

The cytosolic IκBα protein expression remained unchanged throughout the study period (Fig. 3e and Fig. 4e). The cytosolic expression of ubiquitinated proteins was unchanged after immobilization (Fig. 3f). During retraining, an interaction effect was seen (Fig. 4f, *p* = 0.042). However, the post hoc tests could not reveal any significant differences.

## Discussion

The primary finding was that the gross average myofibrillar breakdown rate was lower during 2 weeks of immobilization than during 2 weeks of subsequent retraining, whereas NSAID treatment had no impact on the myofibrillar breakdown rate. Moreover, muscle degradation signaling in the cytosolic and nuclear subcellular fractions differed between the measured time points, and correspondingly, the signaling was unaffected by NSAID treatment.

The present method of measuring muscle protein breakdown rate allowed us to determine a gross average over a 2-week period with the subjects staying in their usual living environments, hence reflecting real-life situations. Previously, the influence of physical inactivity on MPB has been measured in standardized laboratory setups [14, 42, 44]. However, to translate these acute “snap shot” measurements into the long-term changes taking place during periods of free-living muscle unloading the momentary measures of protein breakdown rates do not fully depict how changes in muscle protein breakdown rates underlines changes in muscle mass over time. The present method included both post-prandial and post-absorptive periods as well as periods of sleep, which makes it more representative of the actual myofibrillar breakdown rate during a given intervention and a better exploratory parameter for understanding the mechanisms underlying changes in muscle mass [11]. During the first 2 weeks of immobilization, fluctuations in the breakdown rate may have occurred [1, 44, 49]. A possible increase in MBP from the basal level during the initial days of muscle inactivity may likely have been counterbalanced by a later drop resulting in the reported average rate over the course of the 2-week measuring period [1]. Nevertheless, since no measurement of baseline MPB was included in the present study, it is not possible to say whether the mean MPB during immobilization actually differed from a normal free-living level.

Although it has been demonstrated that MPB increases in the hours/days of recovery following acute resistance exercise [3, 30], it is largely unknown if the mean MPB is similarly affected during longer periods of resistance training. In the present study, a limited number of subjects had the MPB measured (*n* = 7 and *n* = 6 in the Ibu and Plc groups, respectively), and therefore a risk of a type II error should be considered. However, in the two-way ANOVA test, the impact of NSAID appeared nonsignificant; hence, the condition comparison (immobilization versus retraining) becomes rather strong. Therefore, more power (*N* = 13) is present in that comparison, and the finding that the myofibrillar breakdown rate was higher during 2 weeks of unaccustomed rehabilitation resistance training compared to the immobilization period (Fig. 2) is rather strong. It was expected that the MPB would be higher during the retraining period compared to the muscle unloading period, because of the skeletal muscle remodeling processes taking place during early periods of resistance training [8, 9, 50]. Moreover, the present retraining period, following a period of muscle inactivity, could be expected to induce a more pronounced physiological impact than a 2-week period of resistance training applied to normally active muscles, due to the state of muscle atrophy combined with the abrupt and unaccustomed training sessions.

In the following section, we will discuss the findings during the immobilization and retraining periods, separately.

During immobilization, NSAID treatment did neither affect the loss of muscle mass [11], the MPB (Fig. 2), nor degradation signaling (Fig.3). These findings were in contrast to the study hypothesis. Previously, a muscle-protective effect of NSAIDs has been demonstrated in cancer patients and tumor-bearing mice challenged by muscle catabolism [10, 24, 26]. However, the muscle catabolism induced by cancer cachexia may likely differ from the muscle atrophy induced by simple limb immobilization in healthy, older humans. This may at least partly be due to differences in systemic levels of catabolic drivers, such as hypercortisolemia, hypercytokinemia, and insulin resistance that may directly stimulate MPB [1, 34]. Thus, inflammation and muscle degradation presumably plays a more prominent role in cancer-induced cachexia [28, 43], and thus, the underlying muscle-regulatory mechanisms and the effect of NSAIDs may not be directly comparable between cancer patients and healthy older individuals. Somehow in contrast though, dietary fish oil supplementation appears to reduce muscle degradation after 10 days of immobilization even in healthy adult rats [51]. This finding may be explained by the notion that muscle degradation contribute substantially more to the immobilization-induced muscle catabolism in rats than in humans [1, 32].

During retraining, the NSAID treatment did not significantly affect the muscle regrowth in the present study [11]. Previously, both fish oil and NSAID treatment have been shown to impair muscle recovery in rats and mice after periods of muscle inactivity [6, 52], which was associated with a decreased level of muscle inflammation [6]. However, even though muscle damage and infiltration of intramuscular inflammatory cells (e.g., macrophages or leucocytes) were not measured in the present study, it has been shown that NSAIDs do not influence the postexercise production of these inflammatory mediators in human muscle [29, 48]. Moreover, the present resistance training rehabilitation protocol is not comparable to the “normal ambulatory activity” used for muscle recovery in these animal studies [6, 52].

In the present study, protein expression of different signaling molecules involved in muscle protein degradation and inflammation was measured in both the cytosolic and nuclear subcellular fractions (Figs. 3 and 4). The exploration of both breakdown rates by tracer methodology and intramuscular signaling allows a better understanding of the processes and helps the interpretation of the obtained individual results. A general observation was that NSAIDs did not affect the measured signaling molecules, which is in line with the finding that the MPB rate was unaffected by NSAIDs.

Given that inactivity and bed rest have been shown to increase systemic inflammation [7, 22], we hypothesized that NF-kB signaling would increase after 2 weeks of immobilization and that this response would be attenuated by NSAID treatment. However, no changes in cytosolic or nuclear NF-kB p65 were observed in any of the groups, suggesting that local inflammation was not increased by the unilateral leg immobilization. The latter was supported by unchanged IKB-alpha levels. In the absence of an inflammatory response in the placebo group, it is not surprising that NF-kB signaling was unaffected by NSAID treatment. However, we cannot exclude the possibility that an inflammatory response occurred in the initial phase of the 2-week immobilization period.

FoxO3 has been shown to regulate the two major systems of protein breakdown in skeletal muscle, namely, the ubiquitin-proteasome system and the autophagy-lysosomal pathway, through transcription of genes such as MAFbx/atrogin-1, MuRF1, LC3B, and BNIP3 [23, 39]. In a situation of increased muscle protein breakdown, one might therefore expect increased translocation of FoxO3a [17], reflected by decreased cytosolic and increased nuclear levels. Indeed, cytosolic FoxO3a was reduced following 2 weeks of immobilization, but this did not coincide with increased nuclear levels (no change). Accordingly, the cytosolic reduction likely represented attenuated de novo synthesis of FoxO3a. The latter is supported by previous observations of reduced gene expression of FoxO3 following 4 days of immobilization in both young and old men [40]. Although the present reduction in cytosolic FoxO3a immediately after immobilization is somewhat in agreement with the lower myofibrillar breakdown rate during immobilization than during retraining, it should be noted that muscle protein signaling molecules only represent an instant picture of the muscle protein turnover rate at any given time point, which opposites the measured mean MPB rate. Therefore, the measured muscle protein signaling molecules may not necessarily be tightly coupled to the actual muscle protein turnover rate at a given time point.

Ubiquitination of cytosolic proteins, an estimate of the amount of proteins marked for degradation [37], was unchanged in both groups following immobilization. Previously, increased ubiquitination has been observed after 2, but not 14 days of disuse in young individuals [15]. Regardless, results for ubiquitination should be interpreted with care, considering that both the rate of protein ubiquitination and the rate of proteasomal degradation determine the net amount of ubiquitinated proteins at any given time point. Nevertheless, although it only represents an immediate “snap shot,” the measurement of ubiquitinated protein indicates that MPB was not increased from baseline levels after the immobilization period in the present study.

In untrained individuals, resistance exercise causes an inflammatory response [47], as well as increased MPB rates [31]. It has been shown previously that NSAIDs may inhibit training induced increases in gene expression of IL-6 and MuRF-1 in elderly [46]. Therefore, we investigated the effect of NSAIDS on pro-inflammatory signaling and markers of proteolysis in the early and late phase of retraining in the present study. Protein levels of nuclear NF-kB p65 were higher after the first, compared to the last retraining session, and a similar tendency was observed in the cytosolic fraction. This indicates a more pronounced inflammatory response early in the retraining period, when subjects are unfamiliar to the training stimulus. Nevertheless, no effect of NSAIDS was observed, neither on NF-kB, IKB alpha, ubiquitination, nor FoxO3a, in line with the observation that neither FBR nor hypertrophy was affected by the ibuprofen treatment.

## Conclusion

The present study measured the average skeletal muscle myofibrillar breakdown rate in older individuals during daily life periods of immobilization and subsequent rehabilitation training using the deuterated (^2^H_2_O) water–based methodology. The myofibrillar breakdown was lower during immobilization than during retraining but was not affected by NSAID treatment in any of the periods. Moreover, the protein expression of different signaling molecules related to myofibrillar degradation in the cytosolic and nuclear subcellular fractions differed somewhat over the course of the intervention but was likewise unaffected by NSAID treatment.
